# Cytomegalovirus viral load parameters associated with earlier initiation of pre-emptive therapy after solid organ transplantation

**DOI:** 10.1371/journal.pone.0210420

**Published:** 2019-01-25

**Authors:** Sheila Lumley, Cameron Green, Hannah Rafferty, Colette Smith, Mark Harber, James O’Beirne, Gareth Jones, Douglas Thorburn, Aileen Marshall, Tina Shah, Mohamed Zuhair, Emily Rothwell, Sowsan Atabani, Tanzina Haque, Paul Griffiths

**Affiliations:** 1 Centre for Virology University College London Medical School, London, United Kingdom; 2 Institute for Global Health, University College London, London, United Kingdom; 3 Renal Transplant Unit Royal Free Hospital, London, United Kingdom; 4 The Royal Free Sheila Sherlock Liver Centre, Royal Free Hospital, London, United Kingdom; University of St Andrews, UNITED KINGDOM

## Abstract

**Background:**

Human cytomegalovirus (HCMV) can be managed by monitoring HCMV DNA in the blood and giving valganciclovir when viral load exceeds a defined value. We hypothesised that such pre-emptive therapy should occur earlier than the standard 3000 genomes/ml (2520 IU/ml) when a seropositive donor transmitted virus to a seronegative recipient (D+R-) following solid organ transplantation (SOT).

**Methods:**

Our local protocol was changed so that D+R- SOT patients commenced valganciclovir once the viral load exceeded 200 genomes/ml; 168 IU/ml (new protocol). The decision point remained at 3000 genomes/ml (old protocol) for the other two patient subgroups (D+R+, D-R+). Virological outcomes were assessed three years later, when 74 D+R- patients treated under the old protocol could be compared with 67 treated afterwards. The primary outcomes were changes in peak viral load, duration of viraemia and duration of treatment in the D+R- group. The secondary outcome was the proportion of D+R- patients who developed subsequent viraemia episodes.

**Findings:**

In the D+R- patients, the median values of peak viral load (30,774 to 11,135 genomes/ml, p<0.0215) were significantly reduced on the new protocol compared to the old, but the duration of viraemia and duration of treatment were not. Early treatment increased subsequent episodes of viraemia from 33/58 (57%) to 36/49 (73%) of patients (p< 0.0743) with a significant increase (p = 0.0072) in those episodes that required treatment (16/58; 27% versus 26/49; 53%). Median peak viral load increased significantly (2,103 to 3,934 genomes/ml, p<0.0249) in the D+R+ but not in the D-R+ patient subgroups. There was no change in duration of viraemia or duration of treatment for any patient subgroup.

**Interpretation:**

Pre-emptive therapy initiated at the first sign of viraemia post-transplant significantly reduced the peak viral load but increased later episodes of viraemia, consistent with the hypothesis of reduced antigenic stimulation of the immune system.

## Introduction

Cytomegalovirus (HCMV) is an important opportunistic pathogen in solid organ transplant (SOT) patients. In recipients who are HCMV IgG seronegative (R-), primary infection may be transmitted from a seropositive donor (D+). In recipients who are seropositive (R+) for HCMV, the virus may reactivate once they are given immunosuppressive drugs. In addition, a seropositive donor may re-infect a seropositive recipient with a new strain of HCMV.[1] In the presence of immunosuppressive drugs, HCMV in the blood (viraemia) may disseminate to cause end-organ diseases such as hepatitis, pneumonitis, enteritis or retinitis. A high viral load is a prerequisite for causing such end-organ disease and has a sigmoid curve relationship when peak virus load is plotted against risk of end-organ disease.[2, 3] All immunosuppressive drugs work by increasing the viral load with the exception of steroids which interact with viral load to increase the risk of end organ disease associated with a given viral load.[3] The incidence and severity of end-organ disease were both high before transplant centres routinely used one of two strategies for prevention. Patients are either monitored for the presence of viraemia and treated once present with ganciclovir or its prodrug valganciclovir to prevent end-organ disease (“pre-emptive therapy”) or are given valganciclovir from the time of transplant as prophylaxis (“prophylactic therapy”). An analysis by the Cochrane database found 7 randomised controlled trials (RCT) that compared the two strategies, with no significant difference in any of four major clinical outcomes.[4] The results of RCTs of prophylactic therapy show that prophylaxis for 200 days post-transplant is superior to 100 days.[5] However late onset disease, occurring once treatment is stopped, is seen in some patients managed using prophylaxis and may require the use of foscarnet to treat HCMV strains resistant to ganciclovir.[6] Resistant strains may also be seen after pre-emptive therapy. Thus, both pre-emptive therapy and prophylaxis are good strategies recommended in international treatment guidelines, although neither has been able to completely abolish end-organ disease which still occurs, particularly in D+R- patients.[6, 7]

Pre-emptive therapy is used at our institution and provides useful information on the natural history of HCMV.[1] The peak viral load, duration of viraemia and duration of pre-emptive therapy are highest in the D+R- subgroup, intermediate in the D+R+ subgroup and lowest in the D-R+ subgroup, in agreement with their respective high, medium and low risks of HCMV end-organ disease.[1] These differences enable the viral load parameters to be used as robust biomarkers of HCMV activity post-transplant. Now that end-organ disease is uncommon, attention has focussed on using parameters of viral load as a way of assessing immune control of HCMV infection post-transplant. A recent systematic review shows that viral load measurements meet the criteria required of a surrogate marker of HCMV end-organ disease.[8] Having conducted quantitative studies that informed the introduction of pre-emptive therapy, we are keen to continue to refine the strategy to optimise efficacy while minimising drug toxicity. In separate studies, we have used measures of HCMV viraemia as biomarkers to compare antiviral drugs, evaluate a prototype HCMV vaccine and compare viral load cut-offs at which preventive therapy should be initiated.[9–11]

For the current report, we focused on the D+R- subgroup of SOT patients at risk of acquiring primary HCMV infection. To control HCMV replication, these patients must mount an immune response to a new antigen while receiving immunosuppressive drugs. Under the strategy of prophylaxis, the antiviral activity of valganciclovir would be expected to additionally reduce antigen presentation. Thus, potent antiviral drugs given from the time of transplant might impair the ability of the immune system to go through the multiple rounds of cell division required to select high affinity T-cell and B-cell lymphocytes able to control HCMV replication. This could explain why some patients get end-organ disease once prophylaxis is stopped.[6] In contrast, pre-emptive therapy allows antigen presentation to occur [12] and so could be thought of as "endogenous immunisation." Late onset disease is rarely seen after pre-emptive therapy, although this may partly be because some patients develop a second episode of viraemia which is treated pre-emptively.

Until July 2012, pre-emptive therapy was initiated at a viral load of 3000 genomes/ml in all of our patients.[1] Having obtained natural history data[1] to show that D+R- patients have a high probability of developing viraemia while our PCR assay has a negligible occurrence of false positive results (none detected in serial samples from 123 D-R- patients) we altered our protocol to initiate pre-emptive therapy as soon as viraemia became detectable in the D+R- subgroup. We continued to manage the other patient subgroups using the standard protocol and expected that the results of this change would provide further insight into the natural history of primary infection. For example, would it be possible in clinical practice to intervene early so that treatment was begun before viraemia reached 3000 genomes/ml, given[13] the rapid rate of HCMV replication? Would such earlier treatment translate into a lower peak viral load and shorter duration of viraemia? If so, would earlier initiation of pre-emptive therapy still provide sufficient antigen presentation to the immune system so that the number of future episodes of viraemia was not increased? To address these questions, we describe the results of 67 D+R- patients managed consecutively after the change in protocol and compare them with the published results in 74 D+R- patients managed under the old protocol.

The objective was to audit the results of this change for patients transplanted up to 30 June 2015 to determine if biomarkers of viral load were reduced in the D+R- subset, compared to the D+R- subset in the earlier cohort. Because the protocol was not changed for the D+R+ and D-R+ subgroups, we hypothesised that their viral load parameters would remain similar to those reported previously.

## Methods

### Patients and surveillance for HCMV DNA

Patients undergoing HCMV surveillance because of allogeneic transplantation of a kidney or liver were studied in a single institution. Our strategy for HCMV surveillance is described elsewhere.[1] Briefly, samples of whole blood were collected twice a week from in-patients and out-patients for the first 60 days post transplant, then once a week with the objective of monitoring HCMV replication for the first 90 days after transplantation. Further samples were collected from HCMV viraemic patients to follow replication episodes through to resolution. In 2012 we published an audit of the viral load biomarker results in 689 kidney or liver patients transplanted between 2002 and 2010. This total included 123 D-R- patients, so the remaining 566 acted as the comparator group for this study.[1] Policies for immunosuppression did not change between these dates and were based on basiliximab, mycophenolate and tacrolimus for renal transplants and tacrolimus, azathioprine or mycophenolate and steroids for liver transplant recipients.

### Change in protocol for initiating pre-emptive therapy

Up until 1 July 2012, pre-emptive therapy was started in any patient whose viral load was equal to or greater than 3000 genomes/ml. On this date, the protocol was changed so that patients in the D+R- subset were treated when their first PCR positive result was obtained, irrespective of its value. To facilitate prompt initiation of pre-emptive therapy, D+R- patients who were due to be discharged home without having become PCR positive were given a 5-day supply of valganciclovir to be taken only if a member of the transplant team telephoned to say that they had become viraemic. In addition to the routine reporting of PCR results through the hospital’s pathology computer, laboratory staff sent an email to clinical transplant teams when a D+R- patient first had a positive PCR result. Treatment was stopped when two consecutive PCR tests reported that HCMV DNA was undetectable as long as at least 21 days of treatment had been received. The cut-off of 3000 genomes/ml was retained for initiating pre-emptive therapy in the remaining patient subgroups.

For all patients, both before and after the change in protocol, episodes of viraemia that occurred after completion of treatment of the initial episode were only treated if the viral load exceeded 3000 genomes/ml.

### Polymerase chain reaction

The real time PCR method used a Taqman probe and the ABI7700 thermal cycler as described elsewhere and was used to monitor all patients in both time periods.[1] The values of 200 and 3000 genomes/ml of whole blood referred to in this paper, convert to 168 and 2520 international units/ml respectively using the WHO international standard.[14]

### Definition of virological parameters

HCMV viraemia was defined as detection of HCMV DNA in whole blood above the assay cut-off (200 genomes/mL). The duration of viraemia was defined as the total number of days on which HCMV DNA was detected, including repeated episodes of viraemia in the same patient. A repeat episode of viraemia was defined as the presence of HCMV DNA in whole blood detectable following the resolution of a previous episode as documented by two consecutive negative samples. Peak viral load was calculated as the highest recorded HCMV value in an individual patient. HCMV end organ disease was defined using internationally agreed criteria including histological demonstration of inclusion bodies and/or positivity for HCMV proteins by immunostaining, with the exception of HCMV retinitis which was diagnosed ophthalmologically.[15] Duration of antiviral therapy was calculated from the first positive HCMV PCR result until a stop date at the second consecutive PCR result where HCMV DNA was undetectable, with a minimum 21 day course. For patients with more than one episode of viraemia, the total duration of treatment was the sum of individual treatment episodes.

### Statistical methods

The primary outcome was to assess the impact of the new protocol on 3 parameters (peak viral load, duration of viraemia and duration of treatment), in the D+R- transplant subgroup before and after the change in antiviral protocol. Differences between peak viral loads were assessed by Student’s unpaired t-test, because the data were normally distributed. Differences between median durations of viraemia and treatment were assessed by Mann-Whitney U test to allow for their non-parametric distribution.

The secondary outcome was the proportion of D+R- patients who had episodes of recurrent viraemia assessed using the chi-squared test. In addition, comparison of the viral outcomes between the two time periods in the D-R+ and D+R+ groups were performed. As there were no changes to the protocol in these two groups, these analyses served to rule out other changes occurring over calendar time between the two protocol periods.

Post-hoc analysis of the proportion of patients who developed HCMV viraemia in the 3 transplant subgroups divided by organ was performed using the two-sided chi-squared test. Comparisons of the viral parameters between the 3 patient subgroups were performed with a one way ANOVA for peak viral load and Kruskal Wallis test for durations of viraemia and treatment.

For all analyses, no allowance was made for multiple comparisons and a two-sided p-value <0.05 was nominally considered as evidence of a statistically significant difference.

### Ethics

This audit of clinical data was intended to determine if the currently used clinical protocol was working as expected as part of a quality assurance and quality improvement process. In accordance with current UK guidelines, ethical approval was not required because this audit includes only retrospective analysis of results collected for clinical management and all treatment decisions pre-dated the analysis of data.[16] The results were pseudo-anonymised so that no patient identifiers were given to the person performing the statistical analysis.

## Results

### Baseline population characteristics

We describe the viral load parameters of a total of 960 solid organ transplant recipients, transplanted between 1989–2015, before (n = 566) and after (n = 394) a change in treatment protocol for the D+R- patients (baseline demographics shown in Table 1).

**Table 1:** Demographic characteristics of the D+R- cohorts before and after change in antiviral protocol. Data are reported number (%) or mean.

	Before protocol change	After protocol change
Total number of patients	74	67
Sex Male Female	– 31 (42%) 43 (58%)	– 50 (75%) 17 (25%)
Mean age at transplant (years)	42	49
Race - Caucasian - Black - Asian - Other - Unknown	– 45 (61%) 3 (4%) 6 (8%) 2 (3%) 18 (24%)	– 57 (85%) 1 (1%) 4 (6%) 2 (3%) 3 (4%)

Viral load parameters were analysed for 426 liver transplant (262 before change, 164 after change in protocol) and 534 renal (304 renal before, 230 after change). Treatment for the D+R+ and D-R+ patients remained the same throughout. The five most common indications for renal transplant before the change in protocol were diabetes, autoimmune renal disease, IgA nephropathy, idiopathic and adult polycystic renal disease. After the change in protocol these were similar: diabetes, autoimmune renal disease, IgA nephropathy, adult polycystic renal disease and idiopathic. The five most common indications for liver transplant before the change in protocol were hepatitis C, alcoholic liver disease, primary biliary cirrhosis, acute liver failure and primary sclerosing cholangitis. After the change in protocol they were similar: hepatitis C, alcoholic liver disease, acute liver failure, primary sclerosing cholangitis and non-alcoholic steatohepatitis.

The HCMV-related characteristics of the patient groups transplanted in the two time periods were similar (Table 2).

**Table 2:** Viraemia and treatment by donor:recipient serostatus, before and after change in pre-emptive D+R- therapy regimen.

Patient group and category		D+R-		D+R+		D-R+	
		Before	After	Before	After	Before	After
	Number	34	36	112	69	116	59
	Viraemia>200 copies/ml	30(88%)	32(89%)	64(57%)	43(62%)	42(36%)	27(46%)
LIVER	Treated (% DR Group)	26(76%)	32(89%)	27(24%)	29(42%)	10(9%)	10(17%)
	Treated (% of viraemics)	26(87%)	32(100%)	27(42%)	29(67%)	10(24%)	10(37%)
	Number	40	31	158	112	106	87
	Viraemia>200 copies/ml	28(70%)	17(55%)	83(53%)	62(55%)	47(44%)	42(48%)
RENAL	Treated (% DR Group)	25(63%)	17(55%)	35(22%)	34(30%)	19(18%)	10(11%)
	Treated (% of viraemics)	25(89%)	17(100%)	35(42%)	34(55%)	19(42%)	10(24%)
	Number	74	67	270	181	222	146
	Viraemia>200 copies/ml	58(78%)	49(73%)	147(54%)	105(58%)	89(40%)	69(47%)
COMBINED	Treated (% DR Group)	51(69%)	49(73%)	62(23%)	63(34%)	29(13%)	20(13%)
	Treated (% of viraemics)	51(88%)	49(100%)	62(42%)	63(60%)	29(33%)	20(29%)

When followed up to 12 months post-transplant under the new protocol, there were no deaths in the D+R- renal patients, none had to return to dialysis, none had HCMV end-organ disease and one had HCMV syndrome. The corresponding figures for the D+R- liver transplant patients were 3 deaths (all non-HCMV), one case of HCMV end-organ disease and no cases of HCMV syndrome.

### Initiation of pre-emptive therapy under the new protocol

13/17 of the viraemic D+R- renal and 18/32 D+R- liver transplant patients treated under the new protocol had viral loads of less than 3000 genomes/ml at initiation of treatment; these viral loads would have been too low to trigger pre-emptive therapy under the previous protocol. The remaining viraemic patients with initial viral loads more than 3000 genomes/ml would have been immediately treated under either protocol. The median viral load on initiation of antivirals for D+R- patients under the new protocol was 1957 (range 216 to 97551 genomes/ml), compared to 11476 (range 3063–128066 genomes/ml) under the previous protocol.

### Peak viral loads in the new protocol compared to the old protocol

**Fig 1:**
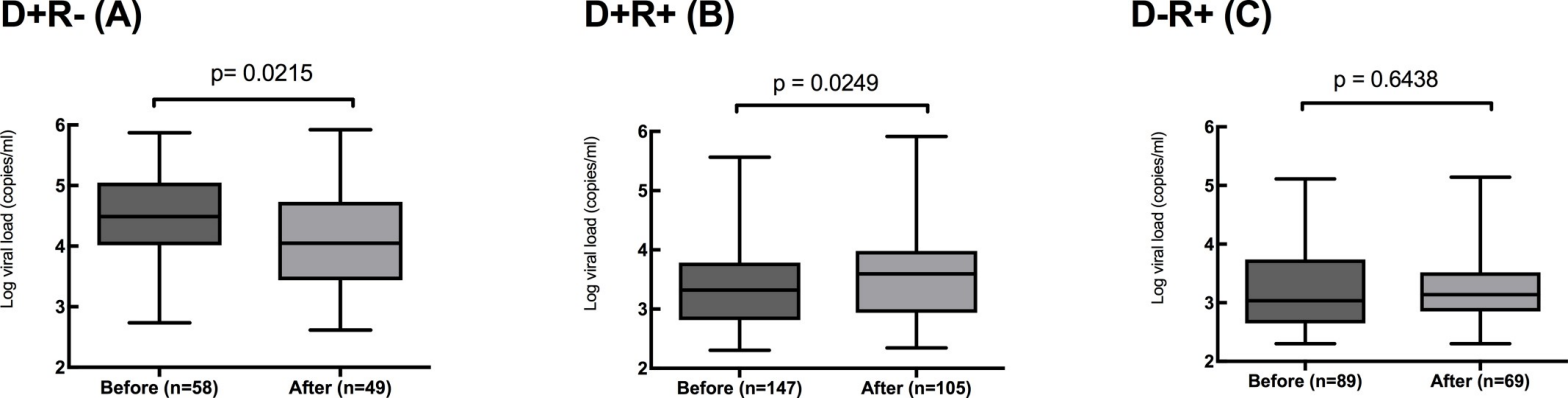
Peak viral loads in the three DR groups D+R- (A), D+R+ (B) and D-R+ (C) transplant recipients, before and after the introduction of the new pre-emptive therapy protocol. Line shows median value, box shows interquartile range and bars indicate range. Non-viraemic patients excluded.

There was a significant reduction for the D+R- subgroup under the new protocol where the median of the peak viral load fell from 30,774 genomes/ml to 11,135 genomes/ml (p = 0.0215, Fig 1A).

The D+R+ subgroup showed a significantly increased median peak viral load from 2103 genomes/ml to 3934 genomes/ml (p = 0.0249, Fig 1B). There was no statistical difference in the values for peak viral load in the D-R+ subgroup. The peak viral loads under the new protocol were significantly different between the three subgroups (p = 0.0004, one-way ANOVA), medians of D+R- 11135 genomes/ml, D+R+ 3934 genomes/ml and D-R+ 1377 genomes/ml)

### Duration of viraemia and duration of treatment in the new protocol compared to the old protocol

**Fig 2:**
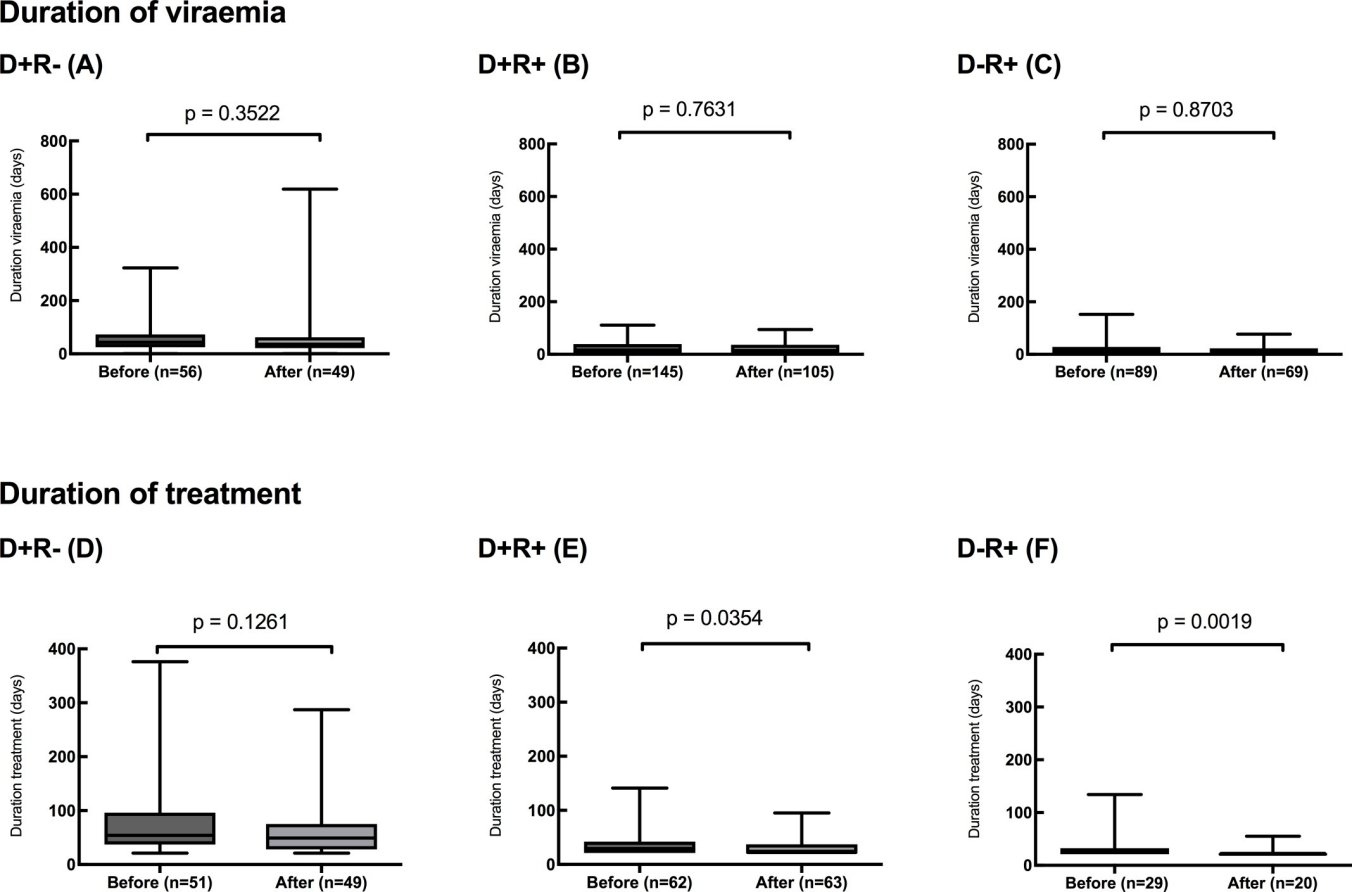
Duration of viraemia (A,B,C) and treatment (D,E,F) in the three DR groups D+R- (A,D), D+R+ (B,E) and D-R+ (C,F) transplant recipients, before and after the introduction of the new pre-emptive therapy protocol. Line shows median value, box shows interquartile range and bars indicate range. Non-viraemic patients excluded.

Panels A-C of Fig 2 show that the total duration of viraemia was not significantly reduced in the D+R- group after switching to the new antiviral protocol, from a median total duration of viraemia of 44 to 36 days (p = 0.352).

Duration of viraemia was significantly different between the three subgroups on the new protocol (p<0.0001, one-way ANOVA), with medians of D+R- 36 days, D+R+ 15 days and D-R+ 11 days.

Panels D-F of Fig 2 show that the total duration of treatment was not significantly reduced in the D+R- group after switching to the new antiviral protocol, from a median total duration of viraemia of 54 to 49 days (p = 0.352).

Significant differences in median duration of treatment were seen between the three subgroups (p<0.0001, one-way ANOVA; 49 days in the D+R- group, 22 days in the D+R+ group and 21 days in the D-R+ group.

### Repeat episodes of viraemia in the new protocol compared to the old protocol

Under the new protocol, the proportion of D+R- patients having a second episode of viraemia after successful treatment of the first was 36/49 (73%), a non-significant increase compared to 33/58 (57%) under the previous protocol (p = 0.0743). Under the new protocol, 26/49 (53%) developed subsequent levels of viraemia high enough to warrant treatment, compared to 16/58 (27%) under the previous protocol (P = 0.0072)

**Fig 3:**
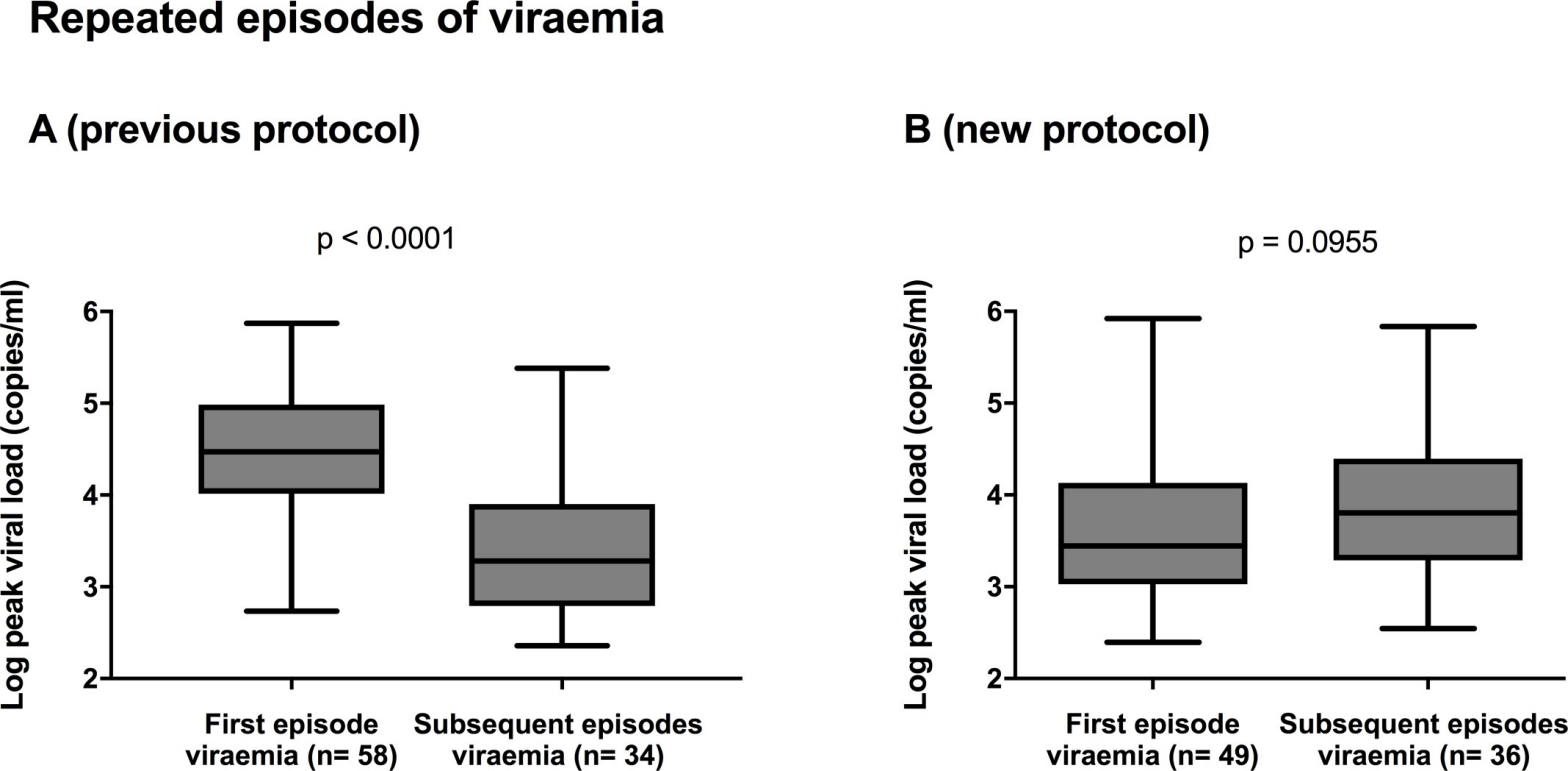
Peak viral loads in D+R- transplant recipients, for the first compared to any subsequent episodes of viraemia, before (A) and after (B) change in protocol. Line shows median value, box shows interquartile range and bars indicate range. Non-viraemic patients excluded.

Among those who had subsequent episodes of viraemia, the peak viral loads in the first peak were not significantly different (p = 0.9) when compared to those in subsequent episodes of viraemia (Fig 3B). Previously peak viral loads had been significantly higher in the first versus subsequent episodes of viraemia (Fig 3A).

## Discussion

We describe the viral load parameters of a total of 960 kidney and liver transplant recipients, before (n = 566) and after (n = 394) a change in treatment protocol for the D+R- patients where treatment for the D+R+ and D-R+ patients remained the same throughout. The change from old to new protocol led to a numerical reduction for all 3 parameters of viral replication in the D+R- patients: 44 to 36 days for days viraemia, 54 to 49 days for days of treatment, and 30,774 to 11,135 genomes/ml for peak viral load, but only the peak viral load changes were statistically significant (p = 0.352, p = 0.352, p<0.0215 respectively). It should be noted that these later episodes are usually smaller than the initial episode so that the peak level is usually derived from the initial episode of viral load.

The remaining patient subgroups were examined in the expectation that no such reduction would be seen, which would act as a control for unrecognised changes in immunosuppression as an explanation for any reduced viral load in the D+R- patients. In practice, there was a significant increase in the peak viral load for the D+R+ subgroup. While this difference remains unexplained and could represent a Type I error, it remains unlikely that the reduction in peak viral load seen in the D+R- patients was due to altered patient management.

The hierarchy of risk for HCMV viral load parameters and risk of end-organ disease has consistently been reported as D+R-, D+R+ and D-R+ in the published literature.[1, 6] We now report a modified management protocol that leads to the peak viral load in the highest risk patients reducing towards that in the D+R+ subgroup. The reduction of median peak viral load from 30,774 genomes/ml to 11,135 genomes/ml (p<0.0215, Fig 1A) was striking. This beneficial outcome was achieved through the combination of a decision to treat early in the infection process coupled with rapid implementation of that decision, facilitated by close liaison between the laboratory and clinical teams. The median viral load upon initiation of treatment was 1,957 genomes/ml with the new protocol versus 11,476 under the previous protocol; a reduction of 83%. However, while the median peak viral load fell from 30,774 to 11,135 genomes/ml, this was only a 64% reduction. This illustrates that changes made to early replication may have non-linear relationships with the outcome parameter and we suggest it represents the momentum inherent in the underlying dynamics of CMV replication.[13]

Given the theories of antigen presentation in pre-emptive versus prophylactic treatment in altering the natural history of HCMV disease, we were interested to see whether earlier initiation of pre-emptive therapy still provided sufficient antigen presentation to the immune system so that the number of future episodes of viraemia was not increased. In fact, we saw an increase in the proportion of patients developing subsequent episodes of viraemia, from 33/58(57%) to 36/49(73%) of patients, p = 0.0743, and in the proportion requiring treatment (16/58; 27% versus 26/49; 53%, p = 0.0072) although there was no change in the overall duration of viraemia or treatment needed. We interpret these results in terms of the antigenic stimulation provided to the immune system and future studies should address this hypothesis by enumerating T-cell and B-cell responses directly. If this hypothesis is confirmed, it would mean that the amount of antigenic stimulus provided to the naïve immune system of a seronegative recipient should not be reduced any further by attempting to initiate pre-emptive therapy at levels of viraemia lower than those employed here. For example, use of a more sensitive PCR method to screen and detect very low levels of viraemia in D+R- transplant patients might provide a paradoxical disadvantage to them in terms of ultimate immune control of HCMV. Given all these results, we will continue to deploy the new protocol to gain more information about the natural history of HCMV and how this could be modified for potential patient benefit. The results also illustrate the potential for evaluating prototype vaccines against HCMV for their ability to prevent second episodes of viraemia in D+R- patients managed with pre-emptive therapy.

## Supporting information

S1 File Spreadsheet containing the data used for the analyses described in this paper. (XLSX)

## Abbreviations

HCMV: human cytomegalovirus
D+: HCMV seropositive donor
R+: HCMV seropositive recipient
SOT: solid organ transplant
